# Reliability, Validity, and Sensitivity of a Specific Agility Test and Its Relationship With Physical Fitness in Karate Athletes

**DOI:** 10.3389/fphys.2022.841498

**Published:** 2022-03-23

**Authors:** Said Ben Hassen, Yassine Negra, Aaron Uthoff, Moktar Chtara, Mohamed Jarraya

**Affiliations:** ^1^Tunisian Research Laboratory Sports Performance Optimization, National Center of Medicine and Science in Sports (CNMSS), Tunis, Tunisia; ^2^Higher Institute of Sport and Physical Education of Ksar Saïd, University of Manouba, Tunis, Tunisia; ^3^School of Sport and Recreation, Sports Performance Research Institute New Zealand (SPRINZ), AUT Millennium, AUT University, Auckland, New Zealand; ^4^Laboratoire Education, Motricité, Sport et Santé, University of Sfax, Sfax, Tunisia

**Keywords:** change of direction speed, combat sports, criterion validity, sport, evaluations

## Abstract

The aim of this study was to investigate the validity and reliability of a developed specific karate agility test (SKAT) and to examine its relationship with physical fitness. A total of 36 karateka voluntarily participated in this study. During two separate sessions, international and national ranked athletes completed the SKAT by performing three changes of direction (CoD): two in a preplanned manner and one in response to a stimulus that was provided by a live tester (Light tester). Assessment of CoD, horizontal jumping ability, 5 and 10 m sprint time, and dynamic balance were also applied during these two occasions. To evaluate SKAT’s construct validity, two groups were recruited based on their karate results: High- vs. low-ranked athletes. Reliability, validity, and sensitivity of the SKAT were examined from the intraclass correlation coefficient (ICC), standard error of measurement (SEM), smallest worthwhile change (SWC), and receiving operator characteristic analysis. Regarding relative and absolute reliability, the ICC of SKAT was excellent at >0.95 and the SEM was <5%. According to the sensitivity analysis, the power to detect small performance changes can be rated as *“good”* in karate athletes (SWC > SEM). The SKAT showed a moderate relationship with the CoD, jumping, sprint, and dynamic balance tests. High-ranked athletes were better than their low-ranked counterparts on SKAT (Cohen’s *d* = 2.00). The area under the receiving operator characteristic curve was 0.76. To sum up, the SKAT is a reliable and valid tool to assess the agility performance of karatekas and can be used by conditioning trainers to detect “true” performance changes.

## Introduction

Karate is an intermittent sport which requires actions with low and high intensity (Chaabene et al., 2012). Performance success in karate depends upon the physical and the physiological attributes of the karateka athlete, along with their technical, tactical, and psychological abilities (Chaabene et al., 2012, 2019). While the technical, tactical, and psychological qualities are not easily measured objectively, the physical and physiological abilities can be objectively evaluated more precisely using standardized methods, providing useful information for coaches.

It is well known that agility is an motor skill important for high-intensity action for combat sports athletes (Chaabene et al., 2012). However, limited studies have addressed the assessment of combat sport athletes’ change of direction (CoD) through sport-specific protocols. Recently, Chaabene et al. (2018) and Chtara et al. (2020) validated new agility tests among taekwondoïstes and fencer athletes, respectively. The two aforementioned studies offer a series of useful indications to assist coaches in the optimization of training programs to achieve high-performance success. Indeed, there is a growing interest toward factors that influence agility performance as well as appropriate testing protocols and training strategies to assess and improve this quality (Paul et al., 2016). Although disparity may exist in the scientific literature, agility is broadly understood to be comprised of both a perceptual decision-making process and a CoD or velocity (Sheppard and Young, 2006). Strength, power, and balance have all been found to be important physical qualities associated with agility performance (Paul et al., 2016). Therefore, high-intensity performance tests (e.g., jumps and sprints) have commonly been used to evaluate explosive performance of karateka (Chaabene et al., 2019). However, these tests lack ecological validity with respect to the specific requirements of karate’s activity. Since success in karate is dependent on the ability to outmaneuver an opponent and deliver more quality strikes than are received (Blazevic et al., 2006), the physical capability to move quickly multi-directionally in response to a stimulus appears to be a key performance trait for karateka (Chaabene et al., 2019).

It has been well established that decisive offensive and defensive karate actions are performed in a limited area, with various rapid displacements of body segments (i.e., forward, backward, and lateral) to perform technical skills reliant on the phosphagen system (Tabben et al., 2013). Most decisive movements last approximately 1–5 s, with 83.8% of actions being executed in less than 2 s and, on average, karateka perform 17 high-intensity decisive movements per match (Tabben et al., 2013). In addition, the decisive movement approach has been found to distinguish between winners and defeated athletes, and 84.4% of the total decisive movements in kumite matches include upper limb techniques to score points (Tabben et al., 2018). Accordingly, agility tests for Karateka should assess performance over a short duration (i.e., no more than 6 s) and include commonly used striking techniques.

Surprisingly, despite the importance of agility in karate, there is no specific karate agility test (to the best of the authors’ knowledge). Therefore, we attempted to fill this knowledge gap with the present experiment. Specifically, this study aimed to (1) investigate the validity, reliability, and sensitivity of a new specific karate agility test and (2) examine its association with different measures of physical fitness (change of direction T-half-test, sprint time, standing long Jump, and dynamic balance test) in karateka athletes.

## Materials and Methods

### Experimental Approach to the Problem

The ecological validity of the specific karate agility test (SKAT) was based on the literature and was designed to reproduce the basic displacement and movement skills of a karate match (e.g., Kizami Tsuki, Gyako Tsuki, and Kiza Mawashi). The criterion validity was ascertained by comparing the SKAT performance with those of a generic CoD T-half-test. The reliability of the SKAT was ascertained by means of test–retest trials separated by 7 days. To test the relationship of the SKAT with proxies of physical fitness performance of karate athletes, sprint time (5 m and 10 m sprint), jumping ability [e.g., standing long jump (SLJ)], and dynamic balance (Y-balance test) tests were selected.

### Participants

A minimum sample size of 36 was determined from an *a priori* statistical power analysis using G^*^Power (Version 3.1, University of Dusseldorf, Germany; Faul et al., 2007). The power analysis was computed with an assumed power of 0.90, an alpha level of 0.05, and a moderate ES (Cohen’s d = 0.50) for the dependent t-test and large ES (Cohen’s d = 1.1) for the independent *t*-test. A total of 36 karateka (20 males and 16 females; age = 20.1 ± 3.1 years; body mass = 66.5 ± 11.1 kg, and height = 177.7 ± 7.9 cm) from the Tunisian national team voluntarily participated in this research. All participants were classified as experienced karateka with a load of eight to nine training sessions per week (during the last 5 years). Each training session lasts 80–90 min. Based on their performance, 25 karateka were classified as high ranked because they competed at the World Championships and had international WKF rankings. The remaining 11 were classified as low ranked because they participated in National and Zone Championships and had low rankings. Written informed parental consent (for those <18 years) and participants’ assent were obtained prior to the start of the study. All karatekas and their legal representatives were fully informed about the experimental protocol and its potential risks and benefits. All procedures were approved by the local ethics committee of the National Centre of Medicine and Science in Sports, Tunisia.

### Procedure

This study was conducted during the first half of the competitive season. Two weeks before the commencement of the study, all athletes attended three orientation sessions. The first session was dedicated to anthropometric measurements (height and body mass). The two other sessions were used for familiarization of all tests. All tests were completed within a two-week period and each test was separated by at least 48 h (two separate phases). For reliability purposes during the first phase, each participant performed the SKAT twice on two separate days. During the second phase, the criterion validity of the SKAT was examined by assessing the correlation with CoD, sprinting, jumping, and dynamic balance performance. All tests were performed during two successive days in the following order: day 1 consisted of the sprint and SKAT tests; day 2 included the jumping and dynamic balance tests. Approximately 20 min of rest was provided between tests to avoid any risk of fatigue.

Tests were preceded by a 10 min of warm-up, including 5 min of running with the remaining time dedicated for static and ballistic stretching, as well as specific submaximal exercises such as kicking, squatting, and jumping. To avoid the risk of diurnal variation on performance, all tests were completed at the same time of day (i.e., 4 pm–6 pm) and under similar environmental conditions (temperature: 19–23°C; relative humidity: 50–60%). All procedures for each test were administered by the same experimenter.

#### Anthropometric Measurements

All anthropometric measurements were taken by a qualified anthropometrist in accordance with standardized procedures of the International Society for the Advancement of Kinanthropometry (ISAK; Stewart et al., 2011). Testing was conducted in a standardized order after careful calibration of the measuring devices. Each karateka’s height (m) and body mass (kg) were assessed to the nearest 0.1 cm and 0.1 kg, using a SECA stadiometer and a SECA weighing scale (SECA Instruments Ltd., Hamburg, Germany), respectively.

#### Specific Karate Agility Test

**Figure 1:**
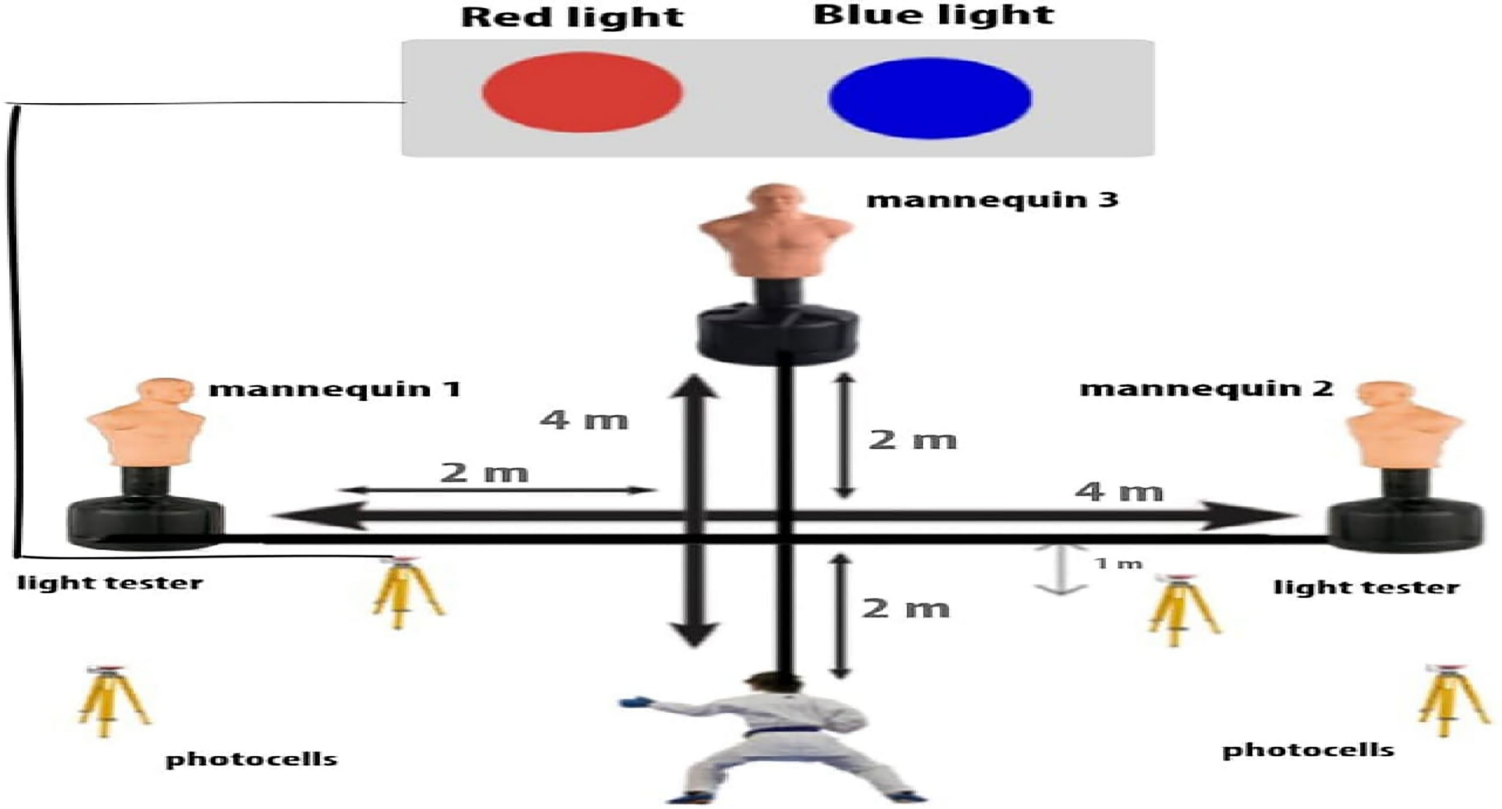
Specific karate agility test.

The dimensions, layout, and movement path through the SKAT are shown in Figure 1. From the start line, participants were required to: (1) move 1 m forward in guard position without crossing feet as quick as possible, where, at this point, the light tester flashed either a red or blue color; (2) athletes continue to the center point; (3) turn toward mannequin 1 or 2 (in regard to the indicated light) by adopting a lateral shift and perform a Kizami Tsuki; (4) move toward mannequin 2 or 1 and perform a Gyako Tsuki; (5) return to the center; (6) move forward in guard position to mannequin 3 and perform a Kiza Mawashi; and (7) move backward to the start/finish line in a guard position (Figure 1). The test was immediately stopped and restarted after 3 min of rest if athletes used karate steps and/or punching technics judged by the evaluators (karate technician/coach) to be technically incorrect. The time needed to complete the test was used as performance outcome and it was assessed with an electronic timing system (Brower Timing Systems, Salt Lake City, United States). The between-trial recovery time was 3 min. The best performance out of three trials was used for further analysis.

#### Change of Direction T-Half-Test

The T-half-test was used to determine speed with directional changes, including forward sprinting, left and right shuffling, and back-pedaling. As outlined by Sassi et al. (2009), subjects started in a split stance with their lead foot 0.30 m behind the starting line A. Subjects sprinted forward to cone B and touched its base with their right hand. Facing forward and without crossing feet, they shuffled to the left to cone C and touched its base with their left hand. Then, they shuffled to the right to cone D, touching its base with their right hands. They next shuffled back to the left to cone B and touched its base. Finally, they ran backward as quickly as possible to line A. Those who crossed one foot in front of the other, failed to touch the base of the cone, and/or failed to face forward throughout had to repeat the test. The time needed to complete the test was used as performance outcome and it was assessed with an electronic timing system (Brower Timing Systems, Salt Lake City, UT, United States). The between-trial recovery time was 3 min. The best performance out of three trials was used for further analysis. The intraclass correlation coefficient (ICC) for test–retest trials was 0.92.

#### Sprint Time

The performance of 0–5 m and 0–10 m linear sprints were recorded using an infrared photocell system (Brower Timing Systems, Salt Lake City, UT, United States) with three single beam photoelectric gates placed at 0 m, 5 m, and 10 m. Players started in a split stance with their lead foot 0.30 m before the first infrared photoelectric gate, which was placed at a height of 0.75 m above the ground (Negra et al., 2017a). The between-trial recovery time was 3 min. The best performance out of three trials was used for further analysis. The ICCs for test–retest trials were 0.96 and 0.97, for 5 m and 10 m, respectively.

#### Standing Long Jump

During the SLJ test, participants stood with their feet shoulder-width apart and in front of a starting line. On the command of “ready, set, go,” participants performed a fast flexion of the legs and downward movement of the arms, before jumping as far as possible in a horizontal direction (Negra et al., 2017b). Participants had to land with both feet at the same time and were not allowed to fall forward or backward (Negra et al., 2017b). The horizontal distance between the starting line and the heel of the rear foot was recorded using a tape measure to the nearest 1 cm. A between-trial rest period of 1 min was allowed. The best out of three trials was recorded for further analysis. The ICC for test–retest reliability was 0.90.

#### Dynamic Balance Test

To measure subject’s dynamic balance, the Y-balance test was used (Negra et al., 2017a). Three tape measures were affixed to the floor, one oriented anterior to the apex (ANT) and the other two were aligned at 135° in the postero-medial (PM) and postero-lateral (PL) directions. Participants stood on the dominant leg on the center of the grid with their barefoot. They were asked to reach with the free limb the farthest distance in the ANT, PM, and PL directions while keeping their single-limb stance. A test trial was judged as invalid and repeated if the participants: (1) did not touch the line with the reach foot while maintaining weight bearing on the stance leg; (2) lifted the stance foot from the center of the grid; (3) missed balance at any point during the trial; (4) did not maintain start and return positions for one full second; or (5) touched down the reach foot to gain considerable support. A between-trial rest period of 1 min was allowed. The best score of three successful attempts expressed as the maximal reach in centimeters for each direction was retained for further analysis with no more than six attempts (Negra et al., 2017a). For normalization purpose, right leg length was measured in cm in a standing position from the inferior aspect of the anterior-superior-iliac spine to the distal-medial malleolus (Negra et al., 2017a). The composite score (CS) was calculated as follows: CS = [(maximum anterior reach distance + maximum postero-medial reach distance + maximum postero-lateral reach distance)/(leg-length × 3)] × 100. The reported test–retest reliability of this test for the three movement directions showed ICCs ranging from 0.84 to 0.89.

### Statistical Analysis

Data analyses were computed using SPSS 24.0 program for Windows (SPSS, Inc., Chicago, IL, United States). The significance level considered in this study was set at *p* < 0.05. Data are expressed as mean and standard deviation. Measures of normality were evaluated using the Shapiro–Wilk’s test. An independent samples t-test was used to determine significant differences in all performance between high-ranked and low-ranked subgroups. The effect size was determined according to Cohen’s d and classified as trivial (<0.20), small (0.20 ≤ *d* ≤ 0.49), medium (0.50 ≤ *d* ≤ 0.79), and large (*d* ≥ 0.80; Cohen, 1988). To determine any learning effect or systematic bias between SKAT and retest scores, a dependent samples t-test was applied. Relative reliability of the SKAT was determined by calculating intraclass correlation coefficient [ICC_(3, 1)_] between the testing occassions. We considered an ICC below 0.40 as poor, between 0.40 and 0.70 as moderate, between 0.70 and 0.90 as good, and ≥ 0.90 as excellent (Chtara et al., 2020). Absolute reliability of the SKAT between testing sessions was analyzed through the standard error of measurement (SEM) expressed as coefficient of variation (CV) and the 95% limit of agreement (LOA) method (Sassi et al., 2009). A SEM of less than 5% was set as the criterion for a good absolute reliability (Negra et al., 2017b). Additionally, to assess operator reliability, Bland–Altman agreement analysis was used. Bland–Altman plots were produced including the 95% CI of the lower and upper LOA, and the mean difference (Sassi et al., 2009). To establish the sensitivity of the SKAT, the smallest worthwhile change (SWC_0.2_) was determined (Chtara et al., 2020). The sensitivity of the test was assessed by comparing the SWC and SEM using the thresholds proposed by Liow and Hopkins (2003). If the SEM was smaller than the SWC, the ability of the test to detect small performance changes was rated *“good.”* If the SEM equaled SWC, then the ability of the test to detect small performance changes was considered *“satisfactory.*” However, in case the SEM was greater than the SWC, the capacity of the test to detect small performance changes was rated *“marginal.”* The minimal detectable change at the 95% confidence interval (MDC_95_) was calculated according to the following formula: MDC_95_ = SEM × 2.77 (Beckerman et al., 2001; Lexell and Downham, 2005). Discriminant validity was established from the receiving operator characteristic (ROC) curve analysis (Chtara et al., 2020). According to Deyo and Centor (1986) an area under the ROC curve >0.70 is considered to indicate acceptable discriminant validity of the test. Pearson’s correlation was used to determine the association between SKAT and the other physical performance tests. Coefficients of determination (*R*^2^) were used to determine the amount of explained variance between tests. The coefficient of determination was calculated as 1 minus the sum of squared regression divided by the total sum of squares (Nakagawa et al., 2017). Hopkins (2000) has suggested that an absolute correlation coefficient of 0–0.1 is considered “trivial,” one of 0.11–0.33 “small,” 0.31–0.5 = “moderate,” 0.51–0.7 = “large,” 0.71–0.9 = “very large,” 0.9–0.99 = nearly perfect,” and 1 = “perfect.”

## Results

### Reliability and Sensitivity of the Specific Karate Agility Test

**Figure 2:**
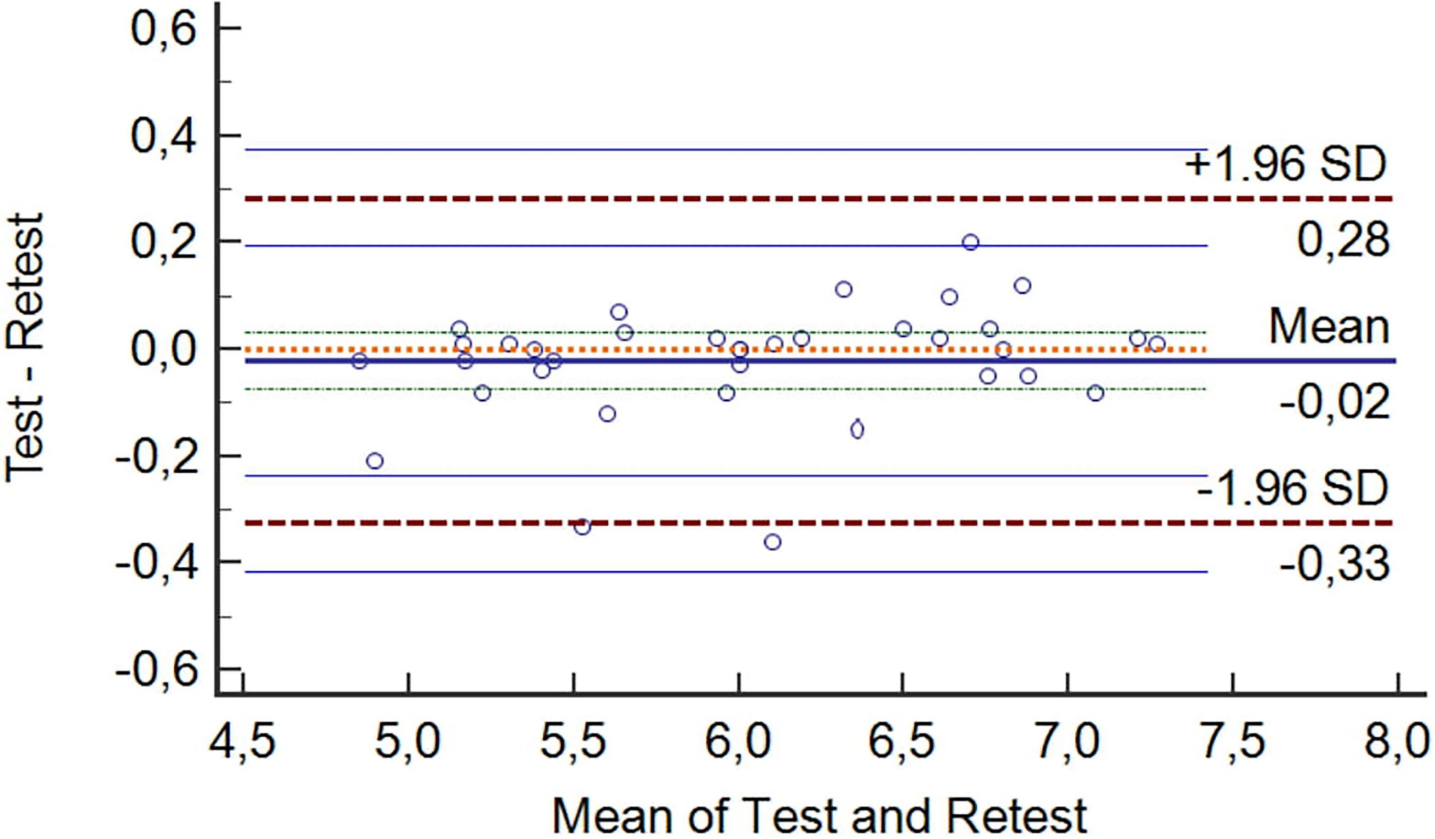
Bland and Altman plots for the specific karate agility test–retest performances.

The findings revealed no significant difference between the SKAT-retest performance (*t* = 0.92; *df* = 35; *p* = 0.60; ES = 0.10). The reliability analyses of the SKAT are mentioned in Table 1. The findings suggest high reliability of the SKAT. Specifically, ICC values were well above 0.90, while SEM values expressed as CV were < 5%. In addition, Bland–Altman plot of first versus second test scores was shown in Figure 2. The mean difference (bias) ± the 95% limits of agreement was 0.02 ± 0.16 s. The ability of the SKAT to detect small performance changes can be rated as *“good,”* given that SWC_0.2_ > SEM. The MDC95 value showed in this study was 0.25 s or 4.18%.

**Table 1:** Reliability results of SKAT.

Parameters	Trial 1 (s)	Trial 2 (s)	ICC [95% CI]	TEM (s)	TEM (%)	SWC_0.2_ (s)	MDC_95_	MDC_95_%
SKAT	6.01 ± 0.73	6.03 ± 0.68	0.98 [0.97–0.99]	0.09	1.5	0.14	0.25	4.18

CI, confidence interval; s, second; SKAT, specific karate agility test; ICC, intraclass correlation coefficient; TEM, typical error of measurement; and SWC, smallest worthwhile change.

### SKAT Performance Between High- and Low-Ranked Athletes

**Figure 3:**
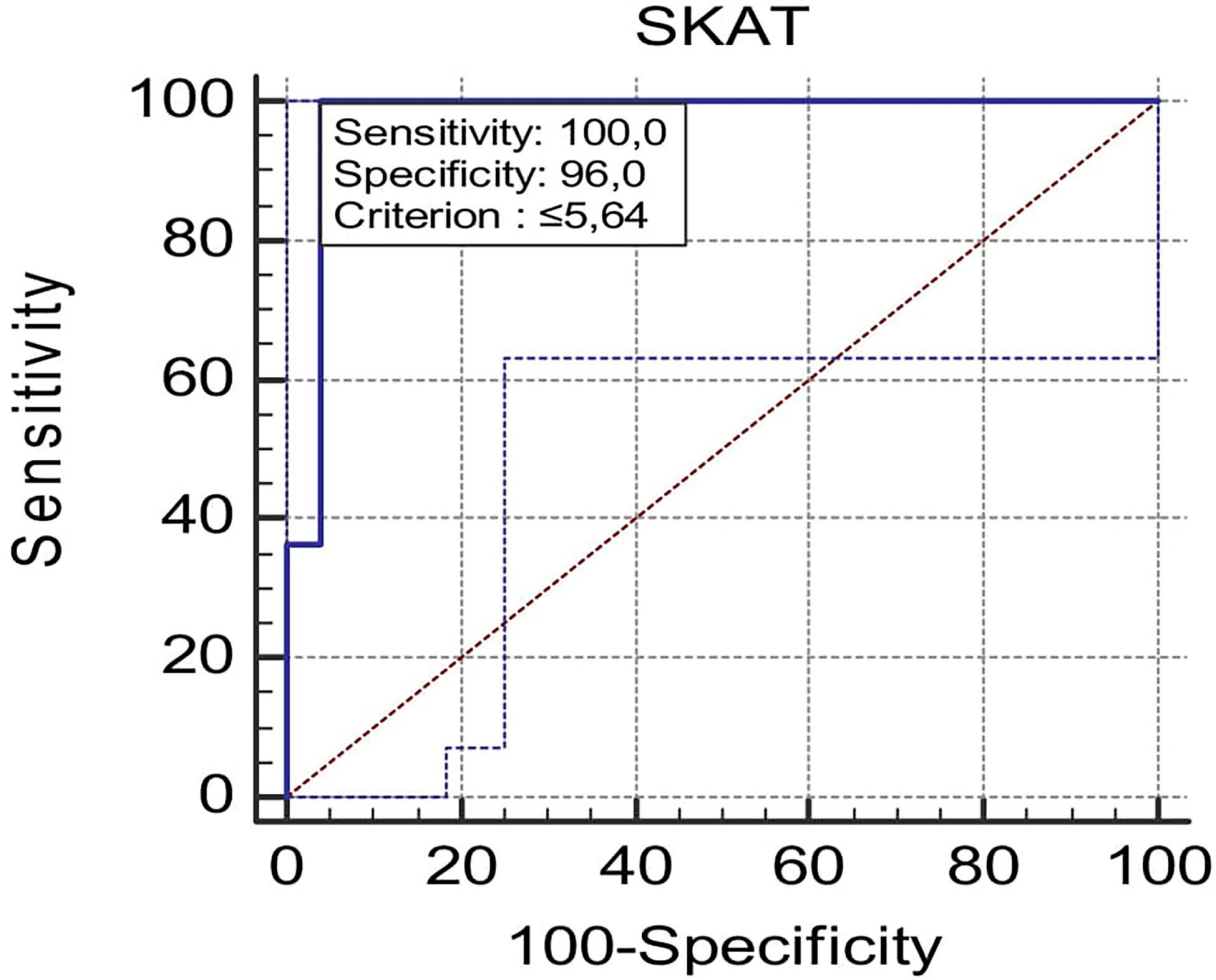
Receiver operating characteristics curve for the SKAT between international and national karateka athletes.

High-ranked karateka revealed greater (*p* < 0.05; ES = 2.00) SKAT performances (5.40 ± 0.36 s) relative to their low-ranked (6.20 ± 0.58 s) counterparts (Table 2). Discriminant validity revealed an AUC of 0.76 (95% CI: 0.86–1.00; *p* < 0.05). The resulting cutoff for the SKAT performance was <5.64 s (Figure 3).

**Table 2:** Differences between high- and low-ranked karateka.

Parameters	High-ranked karatekas	Low-ranked karatekas	Independent Sample *t*-test (95% CI)	Value of *p* (ES)
SKAT	5.40 ± 0.36	6.20 ± 0.58	4.185 (0.41 to 1.18)	<0.001 (1.657)

CI, confidence interval; s, second; and SKAT, specific karate agility test.

### Association Between the SKAT and Measures of Physical Fitness

The findings from the correlational analysis can be found in Table 3. The results revealed a moderate positive relationship (*r* = 0.57, and *R*^2^ = 0.32%) between SKAT performance and the CoD T-half-test. In addition, the findings showed a moderate positive correlation between SKAT and 5 m and 10 m sprint performance (*r* = 0.54–0.58), and a moderate negative relationship with SLJ and dynamic balance test (*r* = −0.52 to −0.58).

**Table 3:** Means ± SD of athletes performances, correlation coefficient (r), and probability values of *p* between KCoDST and the CoD T-half-test (T-half-test), standing long jump (SLJ), sprint test (5 m and 20 m), and composite score (CS) of the Y-balance test.

Test	Mean ± SD	*r*	*p*
SKAT (s)	6.03 ± 0.68	–	–
T-half test (s)	6.22 ± 0.44	0.50	<0.001
SLJ (m)	2.23 ± 0.33	−0.58	<0.001
5 m sprint (s)	1.07 ± 0.11	0.58	<0. 01
10 m sprint (s)	1.99 ± 0.10	0.54	<0. 01
Y-balance Test (CS %)	102.76 ± 6.83	−0.52	<0.01

## Discussion

The purpose of this study was to examine the reliability, validity, and sensitivity of a new specific karate agility test among competitive karateka. The main findings of this study indicated that: (i) SKAT demonstrates a high test–retest reliability (i.e., stable test–retest outcome), useful (i.e., able to detect small changes in performance), and is valid (i.e., significant relationship with CoD T-half-test); (ii) the SKAT showed moderate associations with all the selected measures of physical fitness. These findings are practically meaningful as the SKAT may be used to reliably and accurately track karateka’s performance over time to determine the effectiveness of a training program.

Establishing the error variance of a test enables the quantification of within-test variation (i.e., individual differences on a given day) and test–retest variation (i.e., differences in performance between days; Miaofen and Li-Hua, 2002). Quantifying these reliability metrics of a performance test is the first step to informing coaches of whether it can be used to consistently monitor athlete performance over time. The results of this study showed high relative and absolute reliability of the SKAT (ICC = 0.97 and SEM <5%, respectively). Another common criterion to verify absolute reliability of a test was the Bland and Altman method (Sassi et al., 2009). In these analyses, both bias and random error were found to be low, resulting in a good reliability.

The current findings of high relative and absolute reliability are in line with other combat-specific CoD tests in taekwondo athletes (ICC = 0.97, and SEM = 1.82%; Chaabene et al., 2019). Furthermore, the usefulness of the SKAT was assessed while comparing the SWC0.2 with the SEM. This approach indicates the ability of the SKAT to detect small performance changes. The SEM value of the SKAT was lower than that of the SWC0.2, indicating the good ability of the SKAT to detect small performance change. Therefore, the measurement of total time to perform the SKAT is reliable, and coaches and sports scientists can be confident that performance changes are “true” and not a result of technical or biological variability.

The MDC95 value showed in this study was 0.25 s or 4.18%, indicating that a change in the SKAT performance beyond this value could be considered “real” and reflecting a meaningful performance improvement in karateka. Moreover, significant differences were found between the SKAT performance of high-ranked and low-ranked groups and the discriminant validity, as confirmed by the analysis of the AUC derived from the ROC curves >0.70, suggest that this test may be used to differentiate between high- and low-ranked karateka. While the SKAT is reliable and may be able to distinguish between athlete levels, it is also important to consider its relationship with other commonly used performance tests to determine if it provides unique information related to athletic capabilities specific to karate.

Given the importance of reactive multi-directional ability for karateka (Blazevic et al., 2006), it is important to include tests which truly measure this physical characteristic into a testing battery. The significant correlation between the SKAT and T-half-test performances support the criterion-related validity of the SKAT. While previous studies have utilized change of direction tests, such as the t-test, as an indicator of multi-directional performance in karateka (Koropanovski et al., 2011; Najmi, 2012), these tests do not account for reactive capabilities. Accordingly, in the other combat sports (i.e., Fencing and Taekwondo), agility performances have mainly been assessed *via* a CoD test (Chaabene et al., 2018; Chtara et al., 2020). Since perceptual and decision-making components are integral to reactive agility performance, it is imperative that they be measured concurrently with the physical (i.e., CoD) components (Farrow et al., 2005; Gabbett and Benton, 2009). Accordingly, the SKAT may be considered a more ecologically valid test for assessing agility of karateka than the commonly used CoD tests.

Since strength, power, and balance have all been found to be important physical qualities associated with agility performance (Paul et al., 2016), it was of interest to determine the relationship between the SKAT and these tests. While sprint tests are commonly used as an assessment tool for karateka, this performance metric would seem to have low ecological validity given the 8×8 m competition combat area. Nevertheless, in our study, the statistical calculation showed a moderate correlation between SKAT and 5 m and 10 m sprint performance. According to Chaabene et al. (2019), lower-limb power is considered a fundamental prerequisite in karate especially for the execution of kicking techniques and for CoD performances. The current study found a negative moderate correlation (*r* = −0.57) between the SKAT and the horizontal jumping test. Finally, balance has been considered to play an important role in karate’s activity (Güler et al., 2017; Gauchard et al., 2018; Hadad et al., 2020) and martial arts in general (Chaabene et al., 2018) given that it requires an effective integration of visual, vestibular, and proprioceptive inputs to transition from a dynamic to a static state (Nardone et al., 1997; Young and Willey., 2010; Evangelos et al., 2012). As with CoD, sprint, and jumping performance tests, our findings showed a moderate correlation between SKAT and dynamic balance ability (*r* = −0.52). These findings suggest that the SKAT is associated with the ability to apply horizontal ground-reaction force during sprnting and jumping, as well as dynamic balance capabilities. Therefore, training which aims to improve one of these physical qualities will likely lead to improvements in SKAT performance. However, considering the technical aspects of the sport, relatively more effort should be aimed at enhancing the multi-directional and perceptual components of performance. However, further empirical support is required to substantiate such a position.

Some methodological limitations related to this study warrant discussion. For example, strength and biomechanical test could be induced in future studies to obtain an in depths knowledge regarding their associations with the SKAT. Likewise, it has to be emphasized that correlations are not synonymous with causations. Cross-sectional (i.e., correlational) studies simply show the magnitude of the interrelation between two variables. In other words, significant relationships between sprint time, jumping, balance CoD, and agility performances provide associations between these performance metrics, yet do not establish cause and effect relations. Therefore, the cross-sectional relationships between variables reported in this study should be interpreted with caution. Finally, it is hardly recommended to take into consideration the prospective development of new tests accounting for the high variability of actions/performances in karate.

## Conclusion

The SKAT indicated high validity and reliability in karateka. Additionally, the SKAT showed that it was sensitive to small but meaningful performance changes. Meaning that performance changes on the SKAT can be attributed to “real” changes in performance and not attributed to biological error. Further, the SKAT effectively discriminated between karateka from different rank; therefore, suggesting this test may be useful in sport selection batteries. Moreover, moderate associations were observed between the SKAT and selected measures of physical fitness. Accordingly, strength and conditioning trainers should consider using the SKAT for the purpose of assessing and monitoring the performance of agility among their karateka athletes.

## Data Availability Statement

The raw data supporting the conclusions of this article will be made available by the authors, without undue reservation.

## Ethics Statement

The studies involving human participants were reviewed and approved by Ethics Committee of the National Centre of Medicine and Science in Sports, Tunisia. The patients/participants provided their written informed consent to participate in this study.

## Author Contributions

SB: conceptualization. YN and AU: formal analysis. YN: methodology. YN and SB: writing—original draft. MC and MJ: writing—review and editing. All authors have read and agreed to the published version of the manuscript.

## Conflict of Interest

The authors declare that the research was conducted in the absence of any commercial or financial relationships that could be construed as a potential conflict of interest.

## Publisher’s Note

All claims expressed in this article are solely those of the authors and do not necessarily represent those of their affiliated organizations, or those of the publisher, the editors and the reviewers. Any product that may be evaluated in this article, or claim that may be made by its manufacturer, is not guaranteed or endorsed by the publisher.
